# Supplementary figures and images for: Most endovascular thrombectomy patients have Target Mismatch despite absence of formal CT perfusion selection criteria
Source: PLoS One. 2023 Sep 14;18(9):e0285679. doi: 10.1371/journal.pone.0285679 (PMC10501580; doi:10.1371/journal.pone.0285679)

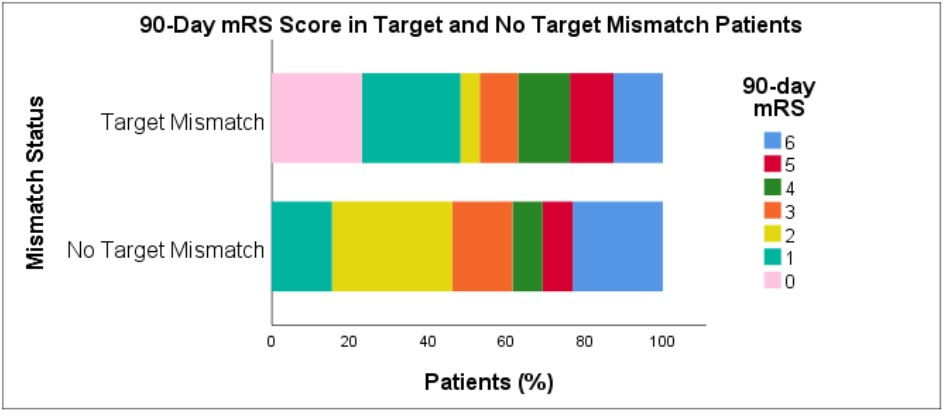

Supplement: S1 Fig — mRS indicates modified Rankin Scale. (TIF) [file pone.0285679.s001.tif]
